# “Does a Good Company Reduce the Unhealthy Behavior of Its Members?”: The Mediating Effect of Organizational Identification and the Moderating Effect of Moral Identity

**DOI:** 10.3390/ijerph18136969

**Published:** 2021-06-29

**Authors:** Byung-Jik Kim, Se-Yeon Choi

**Affiliations:** 1 Department of Business Administration, College of Business Administration, University of Ulsan, Ulsan 44610, Korea; kimbj82@business.kaist.edu; 2 The Institute of Management Research, Seoul National University, Seoul 08826, Korea

**Keywords:** corporate social responsibility, counterproductive work behavior, organizational identification, moral identity, moderated mediation model

## Abstract

In the contemporary business environment where business ethics is critical for organizational performance, the importance of corporate social responsibility (CSR) is increasing. By investigating the mechanism of the effects of CSR on counterproductive work behavior (CWB), the present study suggests that CSR decreases negative employee behavior. Based on social identity theory and context-attitude-behavior framework, this research examines the underlying process and its contingent factor of the association between CSR and CWB. Specifically, this study hypothesizes that CSR decreases CWB by enhancing employees’ organizational identification and that moral identity positively moderates the relationship between CSR and organizational identification. Using three-wave online survey data from 368 employees in Korean firms, this paper tested our hypotheses by conducting moderated mediation analysis with structural equation modeling. The results showed that CSR is negatively related to CWB through organizational identification and that moral identity positively moderates the relationship between CSR and organizational identification. The current study’s findings have crucial theoretical and practical implications in CSR literature.

## 1. Introduction

As corporate ethics has emerged as a critical issue for business, researchers and practitioners have paid attention to corporate social responsibility (CSR) [1,2,3]. While the essence of CSR has been described in different ways, it can be defined as corporate practices and policies that pursue the improvement of economic, social, and environmental performance by satisfying the expectations of the various stakeholders (e.g., employees, consumers, suppliers, communities, governments, environment) [4,5,6,7,8]. Although there have been many studies on the influence of CSR on organizational outcomes, the results remain inconclusive [2,9,10]. Some studies have demonstrated that conducting CSR activities is a strategic “investment” to gain a competitive advantage for a firm [1,11,12]. However, other scholars have criticized that using resources to carry out social responsibilities tends to decrease operational efficiency since it functions as a “cost” [13,14]. To address this controversy, researchers have conducted several works on the intermediating mechanisms and their contingent factors in the CSR-organizational-outcomes link [7,13,15,16,17].

Although several studies have been conducted to investigate the influences of CSR practices on organizational outcomes, several issues still need to be resolved [2,9,10]. First, many existing studies on CSR have primarily focused on the impact of CSR practices on macro-level outcomes (e.g., product quality, corporate reputation, consumer loyalty, financial performance) while relatively underexploring the influence of CSR on individual-level outcomes such as perceptions, attitudes, and employee behaviors [2,9,10]. Considering that the employees are those who not only actually plan and perform the CSR activities within the organization and translate the moral behavior (i.e., CSR) into organizational performance, employees’ reactions (i.e., perceptions, attitudes, and behaviors) toward the CSR practices (i.e., the “micro-foundations of CSR”) must be examined.

Second, although some previous studies have examined the impacts of CSR practices on employees’ reactions, these have mainly focused on their “perceptions and attitudes” such as organizational commitment, job satisfaction, organizational trust, perceived organizational support, work engagement, and organizational identification [3,18,19,20,21,22,23,24], paying less attention to their “behaviors” [2,9,10]. We acknowledge that employees’ perceptions and attitudes are important individual-level outcomes within an organization. However, considering that those perceptions and attitudes are likely to be eventually manifested within the organization in the form of behaviors [9,10,25], we expect that employees’ behaviors are likely to be more closely associated with various organizational outcomes than employees’ perceptions and attitudes. This is why investigating the impacts of CSR on members’ behaviors is required. 

Third and most importantly, existing studies have mainly investigated the impacts of CSR on “positive” outcomes in an organization while underexploring negative ones [9,10]. The literature has demonstrated that CSR activities are likely to enhance the level of positive variables such as organizational commitment, organization identification, organizational trust, and creativity [3,18,19,20,21,22,23,24] while paying less attention to negative variables (e.g., deviant behavior, counterproductive work behavior, withdrawal behavior, and turnover). According to the review paper of Gond and his colleagues [9], CSR scholars should expand the scope of micro-foundation of CSR into an area of negative outcomes to have more extensive knowledge and understanding of the influences of CSR activities [1,9,22]. Given that organizational life consists of positive and negative aspects that have different intermediating mechanisms in an organization [9,10], it is crucial to examine the impacts of CSR on employees’ negative reactions. For example, Gond and his colleagues [9] suggest that the literature needs to include positive outcomes as well as negative and unhealthy outcomes such as stress, strain, burnout, violence, sabotage, and deviance. Through the attempts, the micro-foundation of CSR literature may provide evidence and explanations about whether CSR practices can decrease the harmful and unhealthy behaviors in an organization [1,9,22]. To be specific and pertinent to the second research gap (i.e., underexploring the employee’s “behaviors”), there have been very few studies conducted on influence of CSR on negative behaviors such as deviant behavior and counterproductive work behavior [2,9,10]. 

Fourth, studies on the impact of CSR on employees’ negative behaviors have not sufficiently explored the underlying mechanisms of the relationship [2,8,9,10,26]. By examining the mediating and moderating factors, we can better understand, predict, and control the relationships between the variables. Given that there is little research on the intermediating mechanisms of how CSR practices affect members’ negative behaviors as well as its contingent factors, investigating the association is meaningful. In addition, identifying the intermediating factors and contingent factors would contribute to resolving the inconclusive relationship between CSR and organizational outcomes [2,9,10]. To obtain a precise understanding of the underlying mechanisms, we used a moderated mediation model that combines the moderation and the mediation structure among the variables.

To complement these issues, this paper explores the underlying mechanisms (mediator and moderator) of the relationship between CSR and employees’ negative behaviors such as counterproductive work behavior (CWB) as an individual-level outcome. CWB can be defined as an employees’ intentional behavior that harms the organization or stakeholders [27,28]. This concept has been known to decrease the quality of various organizational outcomes. From the perspective of occupational health, this concept has been considered as an important variable in an organization because it is an obvious consequence of behavioral strain [27,28].

Although employees’ CWB has drawn much recent attention from organizational scholars [28], few have investigated the influence of CSR on CWB [29].

More specifically, based on social identity theory [30] and context-attitude-behavior framework [31], in this paper we suggest that employees’ organizational identification (OI) may mediate the CSR–CWB link. OI is the degree to which members perceive themselves to be one with their organization. This concept can function as a “root construct” within an organization that improves various important organizational outcomes [30,32,33]. According to the social identity theory, the social self of members is influenced by the characteristics of the group they belong to. The more they feel united with the organization, the more they are committed to pursuing and achieving their group’s goals. Employees with a high level of OI are likely to refrain from actions that impair their organization’s goals. When they perceive that their company actively conducts CSR activities, they may have a positive social self that enhances a psychological attachment to and a sense of unity with their organization, ultimately reducing their CWB [23,34,35].

Moreover, we suggest that CSR activities may not always increase the level of employees’ OI, although the argument that CSR enhances the quality of OI is generally acceptable. Considering that there are many individual, situational, and environmental factors in an organization that affect the reactions of employees toward CSR activities, we can expect various contextual or contingent variables that moderate the CSR-OI link. Among the several contingent factors, we focus on the employee’s moral identity based on value congruence theory [36,37]. According to this perspective, the level of value congruence between an employee and his or her organization would significantly influence the perceptions and attitudes toward the organization [36,37]. Members in an organization are unlikely to be passively influenced by the systems and practices implemented by the company. They tend to actively give meaning to the social responsibility activities that companies carry out based on their perceptions, experiences, and values [38,39]. This interpretation and sense-making process encourages members to perceive and respond to the firm’s CSR activities in different ways. Thus, the impact of CSR practices on OI may be significantly affected by various contextual factors. In this paper, among the potential contingent variables, we focus on the level of an employee’s moral identity, which indicates how important an individual perceives themselves to be a “moral being” in defining themselves [40]. The concept has been known to build the basis of an individual’s value system and self-identity [40,41]. Because CSR activities have an inherently moral nature [1,5,7], an individual with a high level of moral identity is likely to be quite interested in and sensitive to how well their organization performs its social responsibility. If the company they belong to actively and sincerely fulfills its social responsibility, their social self would be much more positive than that of a person with a lower moral identity and will increase their sense of unity with their organization. In contrast, for members with low levels of moral identities, the positive influence of the firm’s CSR activities on an employee’s OI may not be as influential because they do not care about the socially responsible acts of the firm. In other words, the impact of CSR practices on an employee’s OI is likely to depend on the level of moral identity of each member.

In this study, we investigate the effect of CSR activities on employees’ CWB through the mediating role of their OI. Furthermore, we suggest that an employee’s moral identity functions as a critical contingent factor that moderates the relationship between CSR and OI. Utilizing three-wave online survey data from 368 employees in South Korean firms, this paper tested the hypotheses by conducting moderated mediation analysis with structural equation modeling. Our study makes the following contributions. First, this paper investigates the influence of CSR on an employee’s behavior, especially negative behavior (i.e., CWB) rather than their perceptions or attitudes. Second, we emphasize the mediating role of employees’ OI as an individual-level underlying mechanism of the CSR-CWB link from the perspective of the micro-foundation of CSR. Third, we reveal that employees’ moral identity, an individual-level contextual or contingent variable, may moderate the impact of CSR on employees’ OI. Lastly, from a methodological point of view, we attempt to complement the limitations of existing studies based on a cross-sectional research design by taking a longitudinal (i.e., 3-wave time-lagged) approach.

## 2. Theory and Hypotheses

### 2.1. CSR and CWB

Although few studies have directly linked the relationship between CSR practices and employees’ CWB, based on the previous works in the field of business ethics, we can expect that CSR would reduce the level of employees’ CWB [29]. According to the literature, a corporate ethical climate reduces deviant behavior of members in an organization [42,43,44]. To be specific, a caring and supportive climate, which is a sub-dimension of the ethical climate, improves members’ ethical decision-making [44], which is likely to improve employees’ attitude toward ethical issues and their ethical behavior [42,43,44]. Considering that the firm’s act of caring for and supporting various stakeholders is fundamentally ethical, CSR activities can be regarded as ethical [1,5,7]. When a company performs well in its social responsibility, its members are likely to perceive that their organization is ethical with a high-level ethical climate. Then the quality of an employee’s ethical decision can be enhanced, in turn facilitating their ethical behavior as well as decreasing unethical behaviors such as CWB [29,42,43,44]. Therefore, we expect that CSR practices are likely both to increase the quality of employees’ ethical behavior and to decrease the degree of unethical behavior [29,30,31,32,33,34,35,36,37,38,39,40,41,42,43,44,45]. For example, Hur and colleagues [29] reported that if a company performs well in its social responsibilities, the level of organizational civility norms improves, which in turn increases the job calling of its members, eventually reducing customer-directed CWB. Moreover, members of an organization that has a high-level ethical climate feel less negative emotions in the workplace, resulting in fewer negative reactions [46], which in turn reduces their deviant behaviors [47,48].

### 2.2. CSR and OI

Based on the literature of micro-foundation of CSR [2,9,10], we suggest that CSR activities may increase the degree of employee OI [23,49], which functions as an organization’s root construct by explaining the perceptions, attitudes, and behaviors of employees [30]. This concept is closely related to the identity and identification of individual employees. Identity indicates “who I am” and “why I work in a certain organization or context,” while identification means a process of establishing the identity pertinent to a certain object or environment/context. Via identification with their group, individual members are likely to build identity within the group [50]. 

The influence of CSR practices on employees’ organizational identification can be explained by these theories. In the field of the micro-foundation of CSR, two of the most promising perspectives are social identity theory (SIT) [30] and perceived external prestige (PEP) perspective [51]. First, the SIT proposes that an individual member’s self is likely to be affected by the group to which they belong. “Social self” is a kind of self that is established by the impacts of the group. From the perspective of members, the organization may be considered one of the most important groups that they belong to; the organization would then be posited in the center of their social self, which in turn influences their self-concept [35]. When the members perceive that the organization performs CSR activities well, they may believe that they belong to a firm with a good reputation in their society, eventually forming a positive social self. Considering that the organization also provides them with fundamental resources and the basis to gain an improved social self-concept, they are more likely to firmly attach to the organization, which facilitates identification with it [35]. 

Second, PEP perspective bolsters the argument that CSR practices may increase the level of employee OI. PEP can be defined as the members’ perceptions of how outside people would evaluate the group they belong to [51]. This is not about how members judge their own group by themselves but about how the members’ belief about their group is perceived by the outside. The concept is also called as “construed external image” [35] or “perceived organizational prestige of a perceived organization” [52]. In accordance with the PEP perspective, members’ OI may be formed by the perception of how outsiders evaluate the organization. Given that the organization is very important to the members, the external perceptions of the organization critically influence their self-esteem or self-concept [35,52]. Therefore, when a firm actively fulfils its social responsibility, the members are likely to possess positive perceived external prestige. Subsequently, they are likely to experience a sense of pride based on the fact that they belong to a firm that conducts valuable missions in society [35]. This can result in a higher level of OI. Based on these arguments, the following hypothesis is proposed.

**Hypothesis** **1.** *CSR is positively associated with OI*.

### 2.3. OI and CWB

We propose that employee OI would decrease the degree of their CWB. Members with a high level of OI may feel a close connection and unity with the organization, feeling a greater sense of belonging to it, and eventually accepting the organization’s values and purpose (Rousseau, 1998; Scott, 1997). This sense of connection, unity, and belonging not only makes members have more positive attitudes toward their organization [30] but also makes them do their best to achieve the organization’s goals and succeed [53,54]. Several studies have shown that OI is closely associated with organizational citizenship behavior [55,56,57] and cooperative behavior [58,59]. 

However, employee OI not only leads to actions that are consistent with the purpose of the organization but also reduces actions that are in accordance with the direction of the organization’s value [27,34,48,60]. Members who feel that they are one with the organization they belong to tend to show less deviant behavior that harms the organization. They are likely to believe that the growth and development of the organization are closely related to their self-concept, eventually facilitating their own growth and development. These employees would try to reduce behaviors that do not achieve the organization’s goals or that impede its success because they perceive that deviant behaviors and CWB would harm the organization as well as themselves [34,48]. Although there have been many studies showing that organizational identification increases positive behaviors among employees (such as organizational citizenship behavior), to the best of our knowledge, few works have found that OI decreases negative behaviors. For example, Al-Atwi and Bakir [34] found that the identification of members reduces both employees’ CWB pertinent to their organization (CWB-O) as well as behavior pertinent to their individual aspect (CWBI). Based on these arguments, the following hypothesis is proposed.

**Hypothesis** **2.** *OI is negatively associated with CWB.*

### 2.4. Mediating Role of OI between CSR and CWB 

By integrating the aforementioned hypotheses that describe direct associations among CSR, OI, and CWB, we suggest that employee OI mediates the link between CSR activities and CWB. This mediation model theoretically relies on a context-attitude-behavior framework [13,31]. According to the framework, various social contexts in an organization, such as an organization’s systems, norms, practices, and activities, may play a critical role in forming employee attitudes and, in turn, determining their behaviors [13,31,46]. Considering that CSR activities would function as important social context, CSR may significantly influence employee attitude (i.e., OI) and behavior (i.e., CWB).

In the current study, based on the theoretical framework of the context-attitude-behavior approach, we expect OI to function as a mediator in the relationship between CSR and CWB. Many previous works have reported that CSR increases the level of OI [23,49]. Moreover, employee OI is likely to decrease the degree of CWB [34,55,56,57]. Thus, we can infer that employee OI may function as an intermediating attitudinal process in the CSR-CWB link. Based on these arguments, the following hypothesis is proposed.

**Hypothesis** **3.** *OI mediates the relationship between CSR and CWB*.

### 2.5. Moderating Effect of Moral Identity in the CSR-OI Link

As described above, several studies have reported that CSR activities improve employee OI [23,49], but it may be a naïve argument that the relationship is always valid in all situations and contexts. We suggest that the CSR-OI link can differently appear depending on the situations, contexts, and especially the characteristics of the members in an organization. Based on value congruence theory [36,37], we propose that employee’s moral identity may moderate the relationship between CSR and OI. Value congruence can be defined as the degree to which an individual’s values are coherent with ones of the organization [36]. According to the theory, the degree of value congruence between an employee and his or her organization would substantially affect the perceptions and attitudes toward the organization [36,37]. Hoffman and his colleagues [37] demonstrated that the value congruence between person and organization mediated the association between transformational leadership and work group effectiveness. Considering that members in an actual organization are not beings who simply accept and conform to the organization’s systems, norms, and actions, but are likely to actively seek meaning and sense making in the process of work experience [39], the value congruence between an employee and organization is critical to building their perceptions, attitudes, and behaviors. They often interpret and give meaning to the organization’s CSR activities based on their own value systems [38]. Through this process of interpretation and sense making, members react differently to the social responsibilities that companies carry out based on the degree of value congruence between them and their organization. 

To be specific, the positive effect of CSR activities on employee OI would be moderated by their level of moral identity. Since an organization’s CSR practices have an inherently moral nature [1,5,7], an employee’s moral identity is likely to determine how much they value CSR activities and how sensitive they are to them [61,62,63]. A person with a high moral identity is very interested in how well the company to which they belong is fulfilling its social responsibilities and will react sensitively to it. If a firm performs well in its social responsibilities, he or she may perceive that the value he or she pursues is coherent with the one of the organization [36,37]. In this situation, he or she is likely to feel a sense of pride, then having a more positive social self than those with low moral identity, and eventually feeling a greater sense of OI. Furthermore, if the firm they belong to does not properly carry out its social responsibilities, they will be more disappointed than those with low moral identity and have a lower level of OI since they perceive that the value they pursue is not congruent with their organization, feeling less pride. Thus, a high level of moral identity is likely to amplify the degree to which CSR activities increase OI [62,63]. 

However, in contrast, a person with a low moral identity is likely not much interested in the social responsibilities of the company to which they belong. For such a person, the degree to which they are moral does not greatly influence the process of forming their identity. The moral self does not play an important role in defining themselves. Because they are not very interested in justice and ethics, they tend not to take these moral factors into account in decision-making [41,62,63]. Therefore, it is not very important for them how much their organization performs moral behaviors, as they are less sensitive to it. Thus, the degree of CSR practices may not have a significant effect on making the employees feel unity with their organization. When the moral identity of employees is low, the enhancing effect of CSR activities on OI would decrease [62,63].

**Figure 1:**
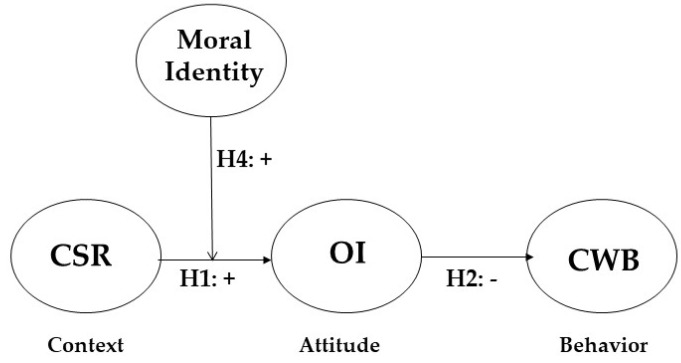
Theoretical Model.

Based on these arguments, we can infer that the enhancing effect of CSR activities on OI may depend on the level of an employee’s moral identity. Moral identity functions as an important contingent or contextual variable which moderates the relationship between CSR and OI. Therefore, this paper proposes the following hypothesis (Please See Figure 1).

**Hypothesis** **4.** *The employee’s moral identity will positively moderate the relationship between CSR and OI*.

## 3. Method

### 3.1. Participants and Procedure

To empirically test the present framework, we collected the survey data from employees in Korean organizations with an online survey system. We conducted data collection via the largest online research firm, which involves the largest Korean research panelists (approximately 1600,000). The participants of the present study were randomly selected by the research firm so that the possibility of biased sampling was reduced. With this online survey system, we collected data from each employee over three different time points at intervals of 4 weeks, preventing the possibility of the same source bias. Through the online system, the research firm could track who responded to the survey, confirming that respondents from time point 1 to time point 3 were the same. The interval among surveys was 4 or 5 weeks. The survey system was open for 2 or 3 days at each time point to provide enough time for respondents. When the system was open, they could access it whenever they wanted.

At Time 1, a total of 770 employees participated. Among those participants, 550 employees responded to our survey at Time 2. At Time 3, we received survey data from 375 employees. After eliminating missing data, data from a total of 368 employees were used for analysis. We believe that this research may diminish the harmful impacts of common method bias (CMB) by measuring each research variable from different time point.

The descriptive characteristics of the final samples are shown in Table 1.

### 3.2. Measures

We asked participants to evaluate the CSR of their organization, their ethical identification, and their demographic characteristics at Time 1. At Time 2, we collected data on organizational identification, and at Time 3, we surveyed counterproductive work behavior (Please See Appendix A). All variables of the current study were assessed with multi-item measures on a five-point Likert scale (1 = strongly disagree, 5 = strongly agree). We calculated the internal consistency of the variables using Cronbach alpha values.

#### 3.2.1. CSR (Collected at Time Point 1, Collected from Employees)

With items of Turker’s CSR scale [24] and other research regarding CSR [23,38], we used a 12-item measure to assess the CSR of each organization. Based on the stakeholders’ perspectives, this scale includes four dimensions classified by various stakeholders: environment, community, employee, and customer. A sample item of the environmental dimension was, “Our company participates in activities that aim to protect and improve the quality of the natural environment.” A sample item of the community dimension was, “Our company contributes to campaigns and projects that promote the well-being of society.” A sample item of the employee dimension was, “Our company policies encourage the employees to develop their skills and careers.” A sample item of the customer dimension was, “The management of our company primarily provides full and accurate information about its products to its customers”. The value of Cronbach’s alpha in the current study was 0.90.

#### 3.2.2. Organizational Identification (Time Point 2, Collected from Employees)

Organizational identification was measured by using five items of Mael and Ashforth [52]. Sample items were, “When someone criticizes my organization, it feels like a personal insult”, “My organization’s successes are my successes”, “When someone criticizes my organization, it feels like a personal insult”, and “When I talk about my organization, I usually say ‘we’ rather than ‘they’”. The value of Cronbach’s alpha in the current study was 0.82.

#### 3.2.3. Counterproductive Work Behavior (Time Point 3, Collected from Immediate Leader)

Drawing on the CWB-checklist [60], we constructed a five-item measure to assess counterproductive work behavior. The participants’ immediate leader or supervisor responded to the CWB questionnaire. The sample items included, “This employee told people outside the job what a lousy place you work for”, “This employee insulted someone about their job performance” and, “This employee purposely worked slowly when things needed to get done.” The value of Cronbach’s alpha in this study was 0.89.

#### 3.2.4. Moral Identity (Time Point 1, Gathered from Employees)

Taking the moral identity items from Aquino and Reed [40], we used a five-item measure to assess the level of moral identity. The scale asks respondents to imagine a person who has nine moral traits (i.e., caring, compassionate, fair, friendly, generous, helpful, hardworking, honest, and kind) and to estimate the extent to which possessing the traits is crucial to the respondent’s sense of himself or herself. The instruction of the measure was, “For a moment, visualize in your mind the kind of person who has the following nine characteristics: caring, compassionate, fair, friendly, generous, helpful, hardworking, honest, and kind. The person with these characteristics could be you or it could be someone else. Imagine how that person would think, feel, and act. When you have a clear image of what this person would be like, answer the following questions.” Sample items were, “It would make me feel good to be a person who has these characteristics”, “Having these characteristics is an important part of my sense of self”, and “Being someone who has these characteristics is an important part of who I am.” The value of Cronbach’s alpha in this study was 0.76.

#### 3.2.5. Control Variables

We controlled for gender, education, position, and tenure of employees to reduce the bias during the estimation process and increased validation because these demographic characteristics tend to influence CWB [63,64,65,66]. All the demographic variables were measured at Time 1.

### 3.3. Analytical Strategy

To validate the hypothesized model, we conducted an analysis using SPSS 21.0 (IBM Corporation, Armonk, NY, USA) and the Amos 21.0 program (IBM Corporation, Armonk, NY, USA). Primarily, we checked demographic characteristics by frequency analysis. To verify the empirical distinctiveness of the measures, we conducted a confirmative factor analysis using the Amos 21.0 program. We examined the relationship between study variables by Pearson correlation analysis. For the hypothesis testing of the current study, we conducted structural equation modeling (SEM) using the Amos 21.0 program. Finally, to examine the mediation effect of organizational identification on the relationship between CSR and CWB, we conducted a bootstrapping analysis [67].

## 4. Results

### 4.1. Descriptive Statistics

Table 2 shows the results of the correlation analysis. The research variables, including CSR activities, OI, and CWB, were significantly associated.

### 4.2. Measurement Model

We conducted confirmatory factor analyses (CFAs) for all 27 items to examine the goodness-of-fit of the measurement model. Because our research model includes four research variables (i.e., CSR, OI, moral identity, and CWB), we considered the discriminant validity of the four variables. Our hypothesized 4-factor model showed a good fit (χ^2^ (df = 139) = 261.106; CFI = 0.961; TLI = 0.952; RMSEA = 0.049). Then we performed a series of chi-square difference tests by consequently comparing the 4-factor model to a 3-factor (χ^2^ (df = 142) = 742.169; CFI = 0.807; TLI = 0.768; RMSEA = 0.107), 2-factor (χ^2^ (df = 144) = 1268.576; CFI = 0.639; TLI = 0.571; RMSEA = 0.146) and a 1-factor (χ^2^ (df = 145) = 1392.340; CFI = 0.599; TLI = 0.528; RMSEA = 0.153) model. The results of the chi-square difference tests indicated that our 4-factor one had the best fit indices among the alternative models. Thus, we suggest that the four variables have a proper level of discriminant validity.

### 4.3. Structural Model

In this paper, we established a moderated mediation model that combines the mediating structure with the moderating one in the CSR-CWB link. In the mediating structure, the CSR-CWB link was mediated by employee OI. In the moderating structure, an employee’s moral identity moderated the influence of CSR on the level of OI.

To check whether there was a multi-collinearity bias between our independent variable and moderator (i.e., CSR and moral identity), we computed the value of variance inflation factors (VIF) and tolerances [68]. The VIF values for CSR and moral identity were 1.01 and 1.01, respectively. Moreover, the tolerance values were 0.99 and 0.99. But the VIF scores were smaller than 10 with tolerance scores above 0.2, so we suggest that CSR and moral identity are relatively free from the issue of multi-collinearity.

#### 4.3.1. Results of Mediation Analysis

To find the best model, we performed SEM analyses and a chi-square difference test with alternative models, including the full mediation model and a partial mediation one. The partial mediation model has a direct path from CSR to CWB. The fit indices of all the alternative models including both full and partial mediation model were good. The results of the chi-square difference test indicates that the full mediation model (χ^2^ (df = 151) = 263.129; CFI = 0.956; TLI = 0.944; RMSEA = 0.045) has a better fit than the partial mediation model (χ^2^ (df = 150) = 262.377; CFI = 0.956; TLI = 0.944; RMSEA = 0.045), meaning that CSR influences the level of CWB indirectly.

**Figure 2:**
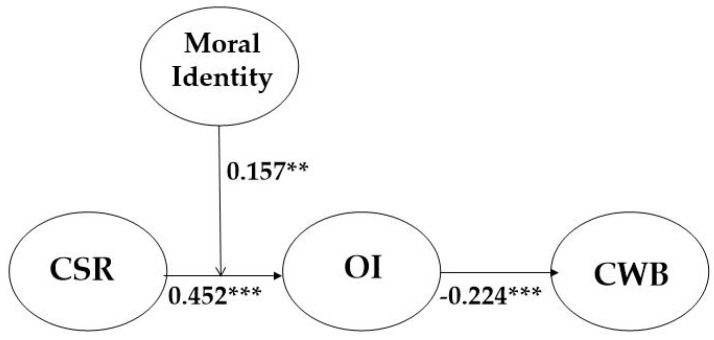
The Result of Coefficient Values of our Research Model (** *p* < 0.01. *** *p* < 0.001).

The control variables (gender, position, tenure, and education level) were statistically non-significant except for gender (β = −0.13, *p* < 0.05). By including the control variables, the research model supported all hypotheses of this paper. CSR increases the level of OI (β = 0.452, *p* < 0.001), supporting Hypothesis 1, and OI diminishes the level of CWB (β = −0.224, *p* < 0.001), supporting Hypothesis 2 (see Figure 2).

#### 4.3.2. Bootstrapping

We performed bootstrapping analyses by using a sample of 10,000 [67] to test Hypothesis 3, a mediation hypothesis. The indirect mediation effect would be significant at the 5% level when the 95% bias-corrected confidence interval (CI) for the mean indirect mediation effect excludes zero [67]. The results demonstrated that the bias-corrected CI for the mean indirect effects on the paths did not include zero (95% CI = [−0.348, −0.079]). Therefore, this paper concludes that Hypotheses 3 is supported. The direct, indirect, and total effects of the paths from CSR to CWB are shown in Table 3.

#### 4.3.3. Results of Moderation Analysis

The moderating effect of moral identity on the CSR-OI link is checked by establishing a moderated mediation model. To create an interaction term, we conducted a mean-centering procedure. Centered variables can not only be used to estimate interaction terms efficiently but also to diminish multicollinearity among the variables [69].

**Figure 3:**
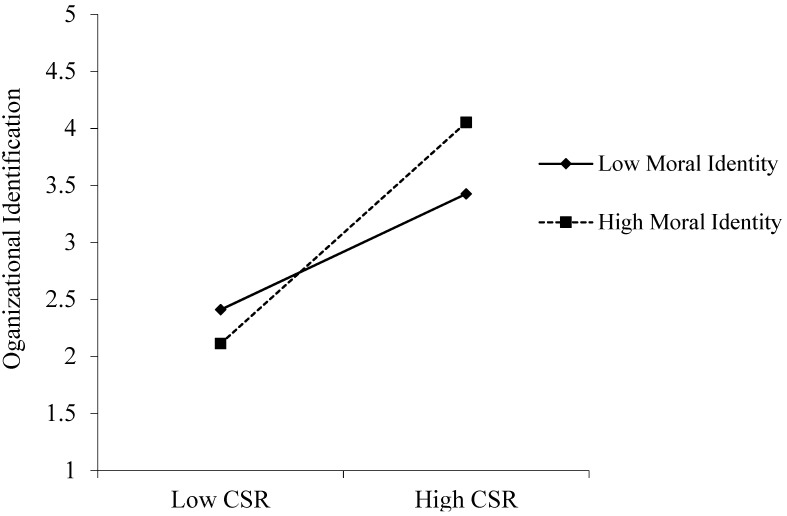
Moderating Effect of Moral Identity in the CSR-OI Link.

The coefficient value of the interaction term was statistically significant (β = 0.157, *p* < 0.01), indicating that moral identity positively moderates the relationship between CSR and CWB. A high-level moral identity would amplify the positive impacts of CSR on employee OI, supporting Hypothesis 4 (Please See Figure 3).

## 5. Discussion

### 5.1. Theoretical Implications

Regarding the theoretical contribution of this study, first, this paper explored the micro-foundation of CSR activities. The literature on CSR has focused mainly on the relationships between CSR and external stakeholders (e.g., shareholders, customers, local communities) and macro-level organizational outcomes (e.g., financial performance, product quality, corporate reputation, consumer loyalty). While the concept of CSR practices is highly associated with a phenomenon at the macro-level, the main actor who plans and executes the CSR activities within the organization and translates it into the macro-level organizational outcomes is the employee. From this perspective, existing studies have not paid sufficient attention to the responses of members (e.g., perceptions, attitudes, behaviors) to CSR activities. This study has a theoretical implication that reveals the micro-level basis for CSR by empirically demonstrating that members of the organization positively respond to CSR practices by enhancing the level of identification with the organization and eventually reduce their CWB.

Second, in this paper, we investigated the influence of CSR activities on members’ negative behavior, CWB. While the literature has mostly focused on the influence of CSR practices on the perceptions and attitudes of members—such as organizational commitment, job satisfaction, organizational trust, perceived organizational support, the meaning of work, and organizational identification—such studies did not pay sufficient attention to CSR’s impacts on their behaviors. Although we acknowledge that members’ perceptions and attitudes are important organizational outcomes, those factors eventually emerge as a form of behavior in an organization. Therefore, the behaviors of members may have a more direct relationship with macro-level organizational performance variables, such as financial performance, than do their perceptions and attitudes. Furthermore, these studies mainly investigated the influence of CSR on employees’ positive behaviors such as organizational citizenship behavior and innovative behavior, relatively paying less attention to their negative behaviors. Considering that positive and negative factors are mixed within an organization and that positive and negative factors have different working mechanisms in the organization [9,10], our attempt in this paper to examine the effect of CSR on CWB should contribute to both CSR and CWB literature.

Third, this research examined the underlying mechanisms of the impact of CSR on CWB by suggesting mediating and moderating variables in the link. CSR practices are likely to enhance the quality of an employee’s social identity. This improved social identity increases their level of identification with the organization, which in turn decreases their negative behaviors (i.e., CWB). Thus, the fulfillment of corporate social responsibility may positively contribute to the level of organizational outcomes in a way that enhances the sense of identification with their organization by improving the quality of employees’ social identity. By explaining why CSR improves organizational performance, this paper contributes to broadening and deepening the scope of CSR literature.

Moreover, this study demonstrated that moral identity of individual members of the organization functions as an important contingent/contextual variable in the CSR-OI link. The degree to which a company’s fulfillment of social responsibilities increases a sense of identification with their organization does not appear to all members in the same way but depends on the level of each member’s moral identity. For example, for an employee whose moral behavior does not have a significant influence on the formation of their identity, no matter how well a company performs its social responsibility, their OI may not be greatly enhanced. Conversely, a member whose moral identity is very important to their self-identity will respond in a way that decreases their level of OI when the company neglects its social responsibility. This study should contribute to the existing research by revealing mediating/regulating variables based on social identity theory to elaborately describe the underlying mechanisms of CSR activities.

### 5.2. Practical Implications

The practical implications of this study are as follows. First, in accordance with the results of this research, corporate executives or leaders need to understand that CSR activities are not a passive and defensive act of simply spending “costs” to fulfill social “duties” [9,10,70]. When a company fulfills its social responsibilities, its members are likely to experience a greater sense of identification and commitment to their organization, which in turn enhances the quality of decision-making and positive behaviors for the organization. To be specific, CSR activities of an organization can significantly decrease the level of employee’s negative and harmful behaviors such as CWB. Considering the substantial and detrimental effects of CWB on various organizational outcomes, the fulfillment of corporate social responsibility can be a kind of meaningful investment to improve organizational performance. This argument is supported by studies that have demonstrated that CSR practices can positively contribute to organizational performance by improving the quality of perceptions, attitudes, and behaviors of members in an organization [2,10].

Second, this study provides insight for the top management team and managers of the organization in that to confirm whether the performance of corporate social responsibility positively affects the members of the organization, they must confirm their sense of unity (i.e., level of organizational identification). The mediating analysis results of this study show that the level of organizational identification of members is mediated in the process of reducing counterproductive task behavior by corporate social responsibility performance. This means that for the corporate social responsibility to exert a positive effect, the level of unity among the members must be increased. An important criterion for judging whether the positive effects of corporate moral behavior are actually appearing within the organization is the level of unity in their organization.

Third, corporate managers need to understand that not all members of the organization respond to corporate social responsibility in the same way. There are individual differences among members. In particular, we showed that the level of moral identity of individual members has a decisive influence on the effect of social responsibility carried out by companies. No matter how well a company carries out its social responsibilities, employees with a low level of moral identity may not have a significant impact on the company’s moral endeavors. Conversely, employees with a high level of moral identity can be very sensitive to the social responsibilities that companies carry out.

### 5.3. Limitations and Suggestions for Future Studies

While this study has theoretical and practical importance, it also has limitations and suggestions for future studies. First, this paper could not objectively measure the degree of CSR activities, even though the degree of objective CSR practices is likely to affect employees’ perceptions, attitudes, and behaviors only through the employees’ interpretation or sense making on the degree of CSR activities perceived as CSR by them. Examining the differential influences of objective CSR and perceived CSR would be meaningful in that the two kinds of CSR concepts indicate different situations of phenomena in an organization. To this end, future studies must collect and analyze data at multiple levels beyond a single-level analysis. Through this methodology, a more elaborate analysis and discussion could be possible by comparing the differential effects of the perceived CSR and objective CSR on employees’ perceptions, attitudes, and behaviors.

Second, in measuring the degree of CSR activities, an independent variable of the study, we could not in this paper sufficiently consider the discriminatory effects of various sub-factors of CSR practices on employees in an organization. As mentioned above, although the targets of corporate social responsibility tend to vary widely (e.g., employees, consumers, suppliers, local communities, the environment, future generations), this research can measure only four dimensions of the CSR activities (i.e., CSR for employees, CSR for customer, CSR for society, and CSR for environment). From the perspective of members of the organization, they are likely to perceive and respond in different ways to the different objectives of CSR activities [20,38]. For example, Farooq and colleagues [20] attempted to verify the differential effects of CSR on members by dividing CSR activities into internal CSR and external CSR. This limitation needs to be addressed by future studies.

Third, although we believe that the fundamental values of CSR activities in Western society would be consistent with ones of Eastern society [71,72], it is reasonable that there are substantial cultural gaps in the member’s interpretation toward CSR practices between the societies. Given that South Korea experienced significantly rapid economic growth, employees in South Korea can be relatively insensitive to moral values in comparison with Western employees [71,73]. However, unfortunately, this research could not fully reflect the issue of cultural differences because it only used data from South Korean employees. Thus, this study should interpret the results in a careful manner [74,75].

## 6. Conclusions

Relying on a context-attitude-behavior perspective, in this paper we investigated the influences of CSR on CWB. Our results demonstrate that CSR activities decrease the level of employees’ CWB by enhancing the level of OI and that an employee’s moral identity functions as a crucial contextual factor in the CSR-OI link. It indicates that employees’ OI is an intermediating process in translating CSR practices into individual-level organizational outcomes (i.e., employees’ CWB). Moreover, employees’ moral identity could facilitate the positive impact of CSR activities on employees’ attitudes (i.e., OI). Although this research has theoretical and practical limitations, our results contribute to CSR literature and CWB literature by describing the underlying process and its contextual factors in the relationship of these variables.

## Figures and Tables

**Table 1:** Descriptive Features of the Sample.

Characteristic	Percent
Gender	
Male	49.7%
Female	50.3%
Age (years)	
20–29	15.2%
30–39	35.9%
40–49	33.1%
50–59	15.8%
Education	
Below high school	8.7%
Community college	27.2%
Bachelor’s degree	87.8%
Graduate degree	12.2%
Position	
Staff	24.2%
Assistant manager	20.9%
Manager	23.9%
Deputy general manager	9.2%
Department/general manager and above director	13.3%
Others	8.4%
Tenure (years)	
Under 5	53.5%
6 to 10	19.9%
11 to 15	13.8%
16 to 20	7.1%
21 to 25	2.1%
Above 25	3.5%
Firm size	
Above 500 members	20.7%
300–499 members	5.7%
100–299 members	15.5%
50–99 members	11.1%
Below 50 members	47.0%
Industry type	
Manufacturing	24.7%
Services	7.1%
Construction	8.7%
Distribution business	12.2%
Information service and telecommunications	7.6%
Education	8.4%
Health and welfare	11.4%
Financial/insurance	3.3%
Real estate	3.8%
Research and Consulting	1.4%
Others	11.4%

**Table 2:** Descriptive statistics.

	1	2	3	4	5	6	7
1. Gender_T2	-						
2. Education	−0.093	-					
3. Tenure_T2	−0.245 **	−0.009	-				
4. Position_T2	−0.408 **	0.213 **	0.266 **	-			
5. CSR_T1	−0.149 **	0.085	0.200 **	0.132 *	-		
6. OI_T2	−0.173 **	0.062	0.160 **	0.184 **	0.400 **	-	
7. CWB_T3	−0.078	−0.077	0.041	−0.045	−0.067	−0.178 **	-
8. Moral Identity_T1	0.056	0.099	−0.109 *	0.050	0.090	0.150 **	−0.184 **

Note: * *p* < 0.05. ** *p* < 0.01.

**Table 3:** Direct, Indirect, and Total Effects of Final Research Model.

Model	Direct Effects	Indirect Effects	Total Effects
CSR –> CWB	0.000	−0.101	−0.101

All values are standardized.

## Data Availability

No new data were created or analyzed in this study. Data sharing is not applicable to this article.

## Appendix A. Measures

### Appendix A.1. Corporate Social Responsibility (Time Point 1, Gathered from Employees)

#### Appendix A.1.1. For the Environmental Dimension

(a) “Our company participates in activities which aim to protect and improve the quality of the natural environment”.
(b) “Our company implements special programs to minimize its negative impact on the natural environment”.
(c) “Our company targets sustainable growth which considers future generations”.

#### Appendix A.1.2. For the Community Dimension

(a) “Our company contributes to campaigns and projects that promote the well-being of society”.
(b) “Our company emphasizes the importance of its social responsibilities to society”.
(c) “Our company actively participates in voluntarily donations to charities and non-governmental organizations”.

#### Appendix A.1.3. For the Employee Dimension

(a) “Management at our company is primarily concerned with employees’ needs and wants”.
(b) “Our company policies encourage employees to develop their skills and careers”.
(c) “Our company supports employees’ growth and development”.

#### Appendix A.1.4. For the Customer Dimension

(a) “Our company respects consumer rights beyond legal requirements”.
(b) “Our company provides full and accurate information about its products to its customers”.
(c) “Customer satisfaction is highly important for our company”.

### Appendix A.2. Organizational Identification (Time Point 2, Collected from Employees)

(a) “When someone criticizes my organization, it feels like a personal insult”.
(b) “My organization’s successes are my successes”.
(c) “When someone criticizes my organization, it feels like a personal insult”.
(d) “When I talk about my organization, I usually say ‘we’ rather than ‘they’”.
(e) “When someone praises my organization, it feels like a personal compliment”.

### Appendix A.3. Counterproductive Work Behavior (Time Point 3, Collected from Immediate Leader)

(a) This employee told people outside the job what a lousy place you work for”.
(b) “This employee insulted someone about their job performance”.
(c) “This employee purposely worked slowly when things needed to get done”.
(d) “This employee complained about insignificant things at work”.
(e) “This employee started an argument with someone at work”.

### Appendix A.4. Moral Identity (Time Point 1, Gathered from Employees)

“For a moment, visualize in your mind the kind of person who has the following nine characteristics: caring, compassionate, fair, friendly, generous, helpful, hardworking, honest, and kind. The person with these characteristics could be you or it could be someone else. Imagine how that person would think, feel, and act. When you have a clear image of what this person would be like, answer the following questions.”

(a) “It would make me feel good to be a person who has these characteristics”.
(b) “Having these characteristics is an important part of my sense of self”.
(c) “Being someone who has these characteristics is an important part of who I am”.
(d) “I would be ashamed to be a person who had these characteristics.”
(e) “I strongly desire to have these characteristics.”

## References

[1] Aguilera R.V., Rupp D.E., Williams C.A., Ganapathi J. (2007). Putting the S back in corporate social responsibility: A multilevel theory of social change in organizations. Acad. Manag. Rev..

[2] Aguinis H., Glavas A. (2012). What we Know and Don’t Know about Corporate Social Responsibility: A Review and Research Agenda. J. Manag..

[3] Müller K., Hattrup K., Spiess S.-O., Lin-Hi N. (2012). The effects of corporate social responsibility on employees’ affective commitment: A cross-cultural investigation. J. Appl. Psychol..

[4] Aguinis H., Zedeck S. (2011). Organizational responsibility: Doing good and doing well. Handbook of Industrial and Organizational Psychology.

[5] Carroll A.B. (1991). The pyramid of corporate social responsibility: Toward the moral management of organizational stakeholders. Bus. Hor..

[6] Carroll A.B. (1999). Corporate social responsibility. Bus. Soc..

[7] McWilliams A., Siegel D. (2001). Corporate social responsibility: A theory of the firm perspective. Acad. Manag. Rev..

[8] Rowley T., Berman S. (2000). A Brand New Brand of Corporate Social Performance. Bus. Soc..

[9] Gond J.-P., El Akremi A., Swaen V., Babu N. (2017). The psychological micro-foundations of corporate social responsibility: A person-centric systematic review. J. Organ. Behav..

[10] Rupp D.E., Mallory D. (2015). Corporate Social Responsibility: Psychological, Person-Centric, and Progressing. Annu. Rev. Organ. Psychol. Organ. Behav..

[11] Greening D.W., Turban D.B. (2000). Corporate Social Performance As a Competitive Advantage in Attracting a Quality Workforce. Bus. Soc..

[12] Porter M.E., Kramer M.R. (2006). Strategy and society: The link between competitive advantage and corporate social responsibility. Harv. Bus. Rev..

[13] Orlitzky M., Schmidt F.L., Rynes S.L. (2003). Corporate Social and Financial Performance: A Meta-Analysis. Organ. Stud..

[14] Van Beurden P., Gossling T. (2008). The worth of values—A literature review on the relation between corporate social and fi-nancial performance. J. Bus. Ethics.

[15] Lai C.S., Chiu C.J., Yang C.F., Pai D.P. (2010). The Effects of Corporate Social Responsibility on Brand Performance: The Me-diating Effect of Industrial Brand Equity and Corporate Reputation. J. Bus. Ethics.

[16] Peng C.-W., Yang M.-L. (2014). The Effect of Corporate Social Performance on Financial Performance: The Moderating Effect of Ownership Concentration. J. Bus. Ethic.

[17] Waddock S.A., Graves S.B. (1997). The Corporate Social Performance-Financial Performance Link. Strateg. Manag. J..

[18] Brammer S., Millington A., Rayton B. (2007). The Contribution of Corporate Social Responsibility to Organizational Commitment. Inter. J. Hum. Res. Manag..

[19] De Roeck K., El Akremi A., Swaen V. (2016). Consistency Matters! How and When Does Corporate Social Responsibility Affect Employees’ Organizational Identification?. J. Manag. Stud..

[20] Farooq O., Rupp D.E., Farooq M. (2017). The multiple pathways through which internal and external CSR influence organiza-tional identification and multi-foci outcomes: The moderating role of cultural and social orientations. Acad. Manag. J..

[21] Glavas A. (2016). Corporate social responsibility and employee engagement: Enabling employees to employ more of their whole selves at work. Front. Psychol..

[22] Glavas A., Kelley K. (2014). The Effects of Perceived Corporate Social Responsibility on Employee Attitudes. Bus. Ethic Q..

[23] Kim H.-R., Lee M., Lee H.-T., Kim N.-M. (2010). Corporate Social Responsibility and Employee–Company Identification. J. Bus. Ethic.

[24] Turker D. (2008). How Corporate Social Responsibility Influences Organizational Commitment. J. Bus. Ethic.

[25] Jones D.A., Rupp D.E., Anderson N., Ones D., Sinangil H., Viswesvaran C. (2016). Social responsibility IN and OF organizations: The psychology of corporate social responsibility among organizational members. Handbook of Industrial, Work, & Organizational Psychology.

[26] Hofman P., Newman A. (2013). The impact of perceived corporate social responsibility on organizational commitment and the moderating role of collectivism and masculinity: Evidence from China. Int. J. Hum. Resour. Manag..

[27] Spector P.E., Fox S. (2006). The Stressor-Emotion Model of Counterproductive Work Behavior. Counterproductive Work Behavior: Investigations of Actors and Targets.

[28] Tepper B.J. (2007). Abusive Supervision in Work Organizations: Review, Synthesis, and Research Agenda. J. Manag..

[29] Hur W.M., Moon T.W., Lee H.G. (2018). Employee engagement in CSR initiatives and customer-directed counterpro-ductive work behavior (CWB): The mediating roles of organizational civility norms and job calling. Corp. Soc. Responsib. Environ. Manag..

[30] Ashforth B.E., Mael F. (1989). Social identity theory and the organization. Acad. Manag. Rev..

[31] Merton R.K. (1968). Social Theory and Structure.

[32] Tajfel H. (1982). Social Psychology of Intergroup Relations. Annu. Rev. Psychol..

[33] Riketta M., Van Dick R. (2005). Foci of attachment in organizations: A meta-analysis comparison of the strength and correlates of work-group versus organizational commitment and identification. J. Vocat. Behav..

[34] Al-Atwi A.A., Bakir A. (2014). Relationships between status judgments, identification, and counterproductive behavior. J. Manag. Psychol..

[35] Dutton J.E., Dukerich J.M., Harquail C.V. (1994). Organizational Images and Member Identification. Adm. Sci. Q..

[36] Edwards J.R., Cable D.M. (2009). The value of value congruence. J. Appl. Psychol..

[37] Hoffman B.J., Bynum B.H., Piccolo R.F., Sutton A.W. (2011). Person-Organization Value Congruence: How Transformational Leaders Influence Work Group Effectiveness. Acad. Manag. J..

[38] Kim B.-J., Nurunnabi M., Kim T.-H., Jung S.-Y. (2019). Does a Good Firm Breed Good Organizational Citizens? The Moderating Role of Perspective Taking. Int. J. Environ. Res. Public Health.

[39] Wrzesniewski A., Dutton J.E., Debebe G. (2003). Interpersonal Sensemaking and the Meaning of Work. Res. Organ. Behav..

[40] Aquino K., Reed A. (2002). The self-importance of moral identity. J. Pers. Soc. Psychol..

[41] Aquino K., Reed A., Thau S., Freeman D. (2007). A grotesque and dark beauty: How moral identity and mechanisms of moral disengagement influence cognitive and emotional reactions to war. J. Exp. Soc. Psychol..

[42] Andreoli N., Lefkowitz J. (2008). Individual and Organizational Antecedents of Misconduct in Organizations. J. Bus. Ethic.

[43] Bulutlar F., Öz E. (2009). The effects of ethical climates on bullying behavior in the workplace. J. Bus. Ethics.

[44] Treviño L.K., Butterfield K.D., McCabe D.L. (1998). The ethical context in organizations: Influences on employee attitudes and behaviors. Bus. Ethics Q..

[45] Moore D.H. (2002). Signaling theory of human rights compliance. Northwestern Univ. Law Rev..

[46] Mulki J.P., Jaramillo J.F., Locander W.B. (2009). Critical Role of Leadership on Ethical Climate and Salesperson Behaviors. J. Bus. Ethic.

[47] Appelbaum S.H., Iaconi G.D., Matousek A. (2007). Positive and negative deviant workplace behaviors: Causes, impacts, and solutions. Corp. Gov. Int. J. Bus. Soc..

[48] Yang J., Diefendorff J.M. (2009). The Relations of Daily Counterproductive Workplace Behavior with Emotions, Situational Antecedents, and Personality Moderators: A Diary Study in Hong Kong. Pers. Psychol..

[49] Carmeli A., Gilat G., Waldman D.A. (2007). The Role of Perceived Organizational Performance in Organizational Identification, Adjustment and Job Performance. J. Manag. Stud..

[50] Ashforth B.E., Harrison S.H., Corley K.G. (2008). Identification in Organizations: An Examination of Four Fundamental Questions. J. Manag..

[51] Smidts A., Pruyn A.T.H., Van Riel C.B. (2001). The impact of employee communication and perceived external prestige on organizational identification. Acad. Manag. J..

[52] Mael F.A., Ashforth B.E. (1992). Alumni and their alma mater: A partial test of the reformulated model of organizational identification. J. Organ. Behav..

[53] Van Dick R., van Knippenberg D., Kerschreiter R., Hertel G., Wieseke J. (2008). Interactive effects of work group and organizational identification on job satisfaction and extra-role behavior. J. Vocat. Behav..

[54] Mathieu J.E., Zajac D.M. (1990). A Review and Meta-Analysis of the Antecedents, Correlates, and Consequences of Organizational Commitment. Psychol. Bull..

[55] Bergami M., Bagozzi R.P. (2000). Self-categorization, affective commitment and group self-esteem as distinct aspects of social identity in the organization. Br. J. Soc. Psychol..

[56] Christ O., Van Dick R., Wagner U., Stellmacher J. (2003). When teachers go the extra-mile: Foci of organizational identification as determinants of different forms of organizational citizenship behavior among schoolteachers. Brit. J. Edu. Psychol..

[57] Van Dick R., Wagner U. (2002). Social identification among school teachers: Dimensions, foci, and correlates. Eur. J. Work. Organ. Psychol..

[58] Dukerich J.M., Golden B.R., Shortell S.M. (2002). Beauty Is in the Eye of the Beholder: The Impact of Organizational Identification, Identity, and Image on the Cooperative Behaviors of Physicians. Adm. Sci. Q..

[59] Tyler T.R., Blader S.L. (2000). Cooperation in Groups: Procedural Justice, Social Identity, and Behavioral Engagement.

[60] Fox S., Spector P.E., Goh A., Bruursema K., Kessler S.R. (2012). The deviant citizen: Measuring potential positive relations between counterproductive work behaviour and organizational citizenship behaviour. J. Occup. Organ. Psychol..

[61] Rupp D.E., Shao R., Thornton M.A., Skarlicki D.P. (2013). Applicants’ and employees’ reactions to corporate social responsibility: The moderating effects of first-party justice perceptions and moral identity. Pers. Psychol..

[62] Reed A., Aquino K.F. (2003). Moral identity and the expanding circle of moral regard toward out-groups. J. Pers. Soc. Psychol..

[63] Skarlicki D.P., van Jaarsveld D.D., Shao R., Song Y.H., Wang M. (2016). Extending the multifoci perspective: The role of supervisor justice and moral identity in the relationship between customer justice and customer-directed sabotage. J. Appl. Psychol..

[64] Penney L.M., Spector P.E. (2005). Job stress, incivility, and counterproductive work behavior (CWB): The moderating role of negative affectivity. J. Organ. Behav..

[65] Samnani A.-K., Salamon S.D., Singh P. (2013). Negative Affect and Counterproductive Workplace Behavior: The Moderating Role of Moral Disengagement and Gender. J. Bus. Ethic.

[66] Sakurai K., Jex S.M. (2012). Coworker incivility and incivility targets’ work effort and counterproductive work behaviors: The moderating role of supervisor social support. J. Occup. Heal. Psychol..

[67] Shrout P.E., Bolger N. (2002). Mediation in Experimental and Nonexperimental Studies: New Procedures and Recommendations. Psychol. Methods.

[68] Brace N., Kemp R., Snelgar R. (2003). SPSS for Psychologists: A Guide to Data Analysis Using SPSS for Windows.

[69] Hayes A.F., Preacher K.J., Hancock G.R., Mueller R.O. (2013). Conditional process modeling: Using structural equation modeling to examine contingent causal processes. Quantitative Methods in Education and the Behavioral Sciences: Issues, Research, and Teaching.

[70] Hou M., Liu H., Fan P., Wei Z. (2016). Does CSR practice pay off in East Asian firms? A meta-analytic investigation. Asia Pac. J. Manag..

[71] Chun J.S., Shin Y., Choi J.N., Kim M.S. (2013). How does corporate ethics contribute to firm financial performance? The role of collective organizational commitment and organizational citizenship behavior. J. Manag..

[72] Eisenbeiss S.A. (2012). Re-thinking ethical leadership: An interdisciplinary integrative approach. Leadersh. Q..

[73] Shin Y., Sung S.Y., Choi J.N., Kim M.S. (2014). Top Management Ethical Leadership and Firm Performance: Mediating Role of Ethical and Procedural Justice Climate. J. Bus. Ethic.

[74] Arnold D.F., Bernardi R.A., Neidermeyer P.E., Schmee J. (2006). The Effect of Country and Culture on Perceptions of Appropriate Ethical Actions Prescribed by Codes of Conduct: A Western European Perspective among Accountants. J. Bus. Ethic.

[75] Nisbett R.E. (2003). The Geography of Thought: How Asians and Westerners Think Differently … and Why.

